# MiR-208b Regulates Rabbit Preadipocyte Proliferation and Differentiation

**DOI:** 10.3390/genes12060890

**Published:** 2021-06-09

**Authors:** Jiahao Shao, Ting Pan, Jie Wang, Tao Tang, Yanhong Li, Xianbo Jia, Songjia Lai

**Affiliations:** 1 College of Animal Science and Technology, Sichuan Agricultural University, Chengdu 611130, China; shaojh1997@126.com (J.S.); wjie68@163.com (J.W.); m18483220592@163.com (T.T.); lyh81236718@163.com (Y.L.); jaxb369@sicau.edu.cn (X.J.); 2 College of Veterinary Medicine, Sichuan Agricultural University, Chengdu 611130, China; panting555666@163.com

**Keywords:** microRNAs, preadipocyte, proliferation, differentiation

## Abstract

microRNAs (miRNAs) play an important role in gene regulation in animals by pairing with target gene mRNA. Many miRNAs are differentially expressed in the adipose tissue, often with conserved expression. In our study, we found that miR-208b expression was observed differently in the preadipocyte differentiation model. When miR-208b was overexpressed in the preadipocyte differentiation model, the overexpressed group displayed higher expression of *PPARγ* and *FABP4*—the markers of preadipocyte differentiation. Oil Red O staining revealed that the count of lipid droplets was increased in the overexpressed group. When the expression of miR-208b was inhibited, the above indicators showed an opposite trend. Moreover, results from both 5-ethynyl-2′-deoxyuridine (EDU) and cell counting kit (CCK) analysis showed that miR-208b promoted the proliferation of preadipocyte. Expression of gene *CSNK2A2*, a direct miR-208b target, was downregulated in the overexpressed group, providing a possible link to multiple signal pathways. Overall, our data indicate that miR-208b play a positive regulatory effect on the proliferation and differentiation of rabbit preadipocyte.

## 1. Introduction

During the past five decades, the prevalence of obesity has rapidly increased, becoming a serious public health issue [1]. Obesity is characterized by the excess accumulation of adipose tissue under the definition of the World Health Organization (WHO) [2]. Overall, adipogenesis is a complex process that is associated with the dynamic expression of multiple transcription factors [3]. Proliferation and differentiation of preadipocyte induce the number of mature adipocyte capable of triglyceride storage, metabolism, and production of adipokines [4]. Rabbit is an ideal experimental animal to study adipogenesis due to its lipid metabolism and obesity-related clinical manifestations similar to those of humans [5,6]. Moreover, compared with other meat, rabbit meat has high digestibility, which is not available in other meat. The rabbit industry plays an important role in the national economy, especially in developing countries [7]. Extensive evidence suggests that meat quality and meat flavor have a great relationship with the fat content of animals [8,9]. Thus, a detailed study of the regulatory mechanisms that underlie rabbit preadipocyte proliferation and differentiation would be expected to contribute to the understanding of adipogenesis and rabbit breeding.

miRNAs have been validated to be able to pair with the 3′ untranslated region (UTR) of target gene mRNA and regulate the target gene at the post-transcriptional level [10]. Extensive work revealed that miRNAs are involved in the regulation of preadipocyte proliferation and differentiation. For example, a previous study reported that miR-143a-3p modulates preadipocyte proliferation and differentiation by targeting MAPK7 [11]. miR-125a-5p promotes 3T3-L1 preadipocyte proliferation, while negatively regulating STAT3 to inhibit 3T3-L1 preadipocyte differentiation [12]. Previous studies have found that miR-208b is a key molecule that poses the regulated function for a variety of diseases including chronic chagas disease cardiomyopathy and acute myocardial infarction [13,14]. Moreover, it is important that miR-208b regulates intracellular fatty acid and glucose metabolism and influences the development of obesity [15]. The above studies revealed that miR-208b may regulate the proliferation and differentiation of preadipocyte, but the specific mechanism remains unclear. Therefore, we investigated the role of miR-208b in rabbit preadipocyte proliferation and differentiation by overexpressing or inhibiting miR-208b.

## 2. Materials and Methods

### 2.1. Animals and Cell Collection

A newborn New Zealand rabbit was used for sampling. Briefly, the experimental rabbit was rubbed all over with alcohol and sacrificed humanely to reduce suffering. The primary preadipocyte was collected in the perirenal area of newborn New Zealand rabbits using the method described earlier [16]. All experiments in the present trial involving animals were performed under the direction of the Institutional Animal Care and Use Committee from the College of Animal Science and Technology, Sichuan Agricultural University, China (DKY-B2019202015). 

### 2.2. Cell Culture

Preadipocyte was maintained in a growth medium (GM) containing 10% fetal bovine serum (FBS, Gibco, Carlsbad, CA, USA) in an incubator (Thermo Scientific, Waltham, MA, USA) at 37 °C and 5% CO_2_ after sterility separation, and the medium was changed every two days. When the cell density reached 80%, it was purified and passage to obtain the third generation of preadipocyte for subsequent experiments. To stimulate preadipocyte differentiation, the third generation of preadipocyte was cultured in differentiation media (DM) containing Dulbecco’s modified Eagle’s medium (DMEM, Gibco, Carlsbad, CA, USA) supplemented with 5% FBS, 0.5 mM dexamethasone (DEX), 0.5 mM 3-isobutyl-1-methylxanthine (IBMX), and 10 mg/mL insulin for three days. After that, the medium was replaced by a maintenance medium (MM) consisting of DMEM with 5% FBS and 10 mg/mL insulin for three days. Subsequently, the medium was replaced with GM containing 5% FBS.

### 2.3. Transfection

When the cell density reached approximately 80% confluence, they were transfected with miR-208b mimic, miR-208b inhibitor, miR-208b negative control (NC), and miR-208b inhibitor negative control (INC) using the lipofectamine 3000 reagent (Invitrogen, Carlsbad, CA, USA), following the manufacturer’s protocol. The above RNA oligo (mimic, inhibitor, NC, INC) was purchased from the Sangon Bioengineering Co., Ltd (Shanghai, China), and the sequences are shown in Table 1. Subsequently, the cell was kept in the transfection reagent for 6 h and then changed to the corresponding DM to induce differentiation. For cell proliferation experiments, transfection was performed as described above when the cell density reached 50%. After 6 h, the medium was changed to GM.

### 2.4. Quantitative Real-Time Polymerase Chain Reaction Analysis

Total RNA from the cell was obtained using the RNAiso Plus Reagent (Invitrogen, Hong Kong, China) according to the standard guidelines. The Agilent 2100 Bioanalyzer system (Agilent Technologies, Carlsbad, CA, USA) was used for quality inspection, and only qualified RNA (A160/A180 = 1.6–1.8, concentration > 200 ng/uL) was used for the later trial. Reverse transcription of mRNA and miRNA was performed using the PrimeScript™ RT reagent Kit with gDNA Eraser (Takara, Dalian, China) and the Mir-X™ miRNA First-Strand Synthesis Kit (Takara) following the manufacturer’s protocol, respectively. Subsequently, qRT-PCR was performed in triplicate using the TB Green™ Premix Ex Taq™ II (Takara) on a CFX96 instrument (Bio-Rad, Hercules, CA, USA), and the relative expression levels of mRNA and miRNA were calculated using the 2^−ΔΔCt^ method. The mRQ 3′ primer in the Mir-X™ miRNA First-Strand Synthesis Kit (Takara) was used and served as a reverse primer for miRNA quantification, and U6 was used as an internal reference. Besides, *GAPHD* was used as an internal reference for mRNA quantification. Detailly, the primer sequences are listed in Table 2.

### 2.5. Protein Extraction and Western Blotting

Total protein from the cell was collected using a commercial Protein Extraction Kit (Sangon, Shanghai, China), following the manufacturer’s protocol. The concentration of the protein was measured using the Bradford protein assay kit (Beyotime, Jiangsu, China), and only protein meeting quality criteria were used for the further trial. Briefly, the protein was resolved on 10% SDS-PAGE and then transferred to a PVDF membrane, followed by sealing of the sealing fluid. The membranes were incubated with the correspondingly primary antibodies for 24 h at 4 °C and subsequently incubated with the secondary antibodies for 2 h. The membranes were subjected to chemiluminescence reagents to detect immunoreactivities. A GelDoc system equipped (Bio-Rad, Hercules, CA, USA) was used to capture images. β-actin protein was used as an internal control.

### 2.6. CCK Assay

Cell counting kit (CCK, GOYOD, Nanjing, China) was used to examine the effect of miR-208b on preadipocyte proliferation. Briefly, the third generation of preadipocyte was seeded in 96-well plates and transfected with miR-208b mimic, inhibitor, NC, and INC at 50% cell density. Six hours later, the medium was changed to GM. After incubation for 24 h, 48 h, 72 h, 96 h, and 120 h, 10 μL of CCK reagent was added to each well and incubated at 37 °C and 5% CO_2_ for 2 h. In living cells, 2-(2-methoxy-4-nitrophenyl)-3-(4-nitrophenyl)-5-(2,4-disulfophenyl)-2h-tetrazolium sodium salt is reduced to orange formazan by dehydrogenase. The number of living cells is proportional to the amount of formazan, the amount of formazan is proportional to the absorbance at 450 nm. Thus, the absorbance of the sample can represent the number of living cells and was measured by a microplate reader (Thermo Scientific, Waltham, MA, USA).

### 2.7. EDU Proliferation Assay

Preadipocyte was grown in 12-well plates. Transfection and medium replacement were performed as described above. After 48 h, the cell was cultured for 2 h in GM containing 100 μL 5-ethynyl-2′-deoxyuridine (EDU, RiboBio, Guangzhou, China). Next, the cell was immobilized and stained according to the company’s instructions. The staining of EDU and Hoechst in the same field were photographed using an inverted fluorescence microscope (Olympus, Tokyo, Japan). Images were analyzed by using image-pro plus 6.0 software (Media Cybernetics, Inc, Rockville, MD, USA).

### 2.8. Oil Red O Staining

Oil Red O staining was performed at room temperature. The cell was washed three times with phosphate-buffered saline (PBS) and fixed in 10% paraformaldehyde for 30 min. Subsequently, Oil Red O was mixed with deionized water at a rate of 3:2 and then added to the stained cell for 1 h. Finally, the above cell was rinsed with PBS until there were no obvious impurities. Images were captured using an inverted microscope (Olympus). Moreover, the count of lipid droplets was measured by using image-pro plus 6.0 software (Media Cybernetics).

### 2.9. Target Genes Prediction and Verification

The target genes of miR-208b were predicted using the online database TargetScan (http://www.targetscan.org/mamm_31/). Gene Ontology (GO) analysis and Kyoto Encyclopedia of Genes and Genomes (KEGG) pathway enrichment analysis were performed using software DAVID 6.7 (http://david.abcc.ncifcrf.gov/home.jsp). Moreover, the MiRWalk database (http://zmf.umm.uni-heidelberg.de/apps/zmf/mirwalk2/) was used to predict the potential site that binds with miR-208b. To validate the binding site, luciferase reporter plasmids (wild-type and mutant 3′ UTR of *CSNK2A2*) were constructed by Tsingke Biotechnology Co., LTD (Tsingke, Chengdu, China). Hela cell was seeded into 24-well plates. The wild-type or mutant plasmids were cotransfected with miR-208b mimic into Hela cell when the cell density reached 70%. Then, luciferase activities were measured using the TransDetect^®^ Double Luciferase Reporter Assay Kit instructions (Transgen, Beijing, China) after 24 h, following the manufacturer’s guides.

### 2.10. Statistical Analysis

All data are presented as means ± SEM. The SPSS 22.0 software (SPSS Inc. Chicago, IL, USA) was used for statistical analysis, and differences between groups were determined by Student’s *t*-test. Moreover, multiple comparisons were performed using one-way ANOVA followed by Dunnett’s posthoc analysis. Two-way ANOVA was performed to compare differences between the two groups for *PPARγ* and *FABP4*. Differences were considered statistically significant at *p* < 0.05.

## 3. Results

### 3.1. Establishment of Rabbit Preadipocyte Differentiation Model

**Figure 1:**
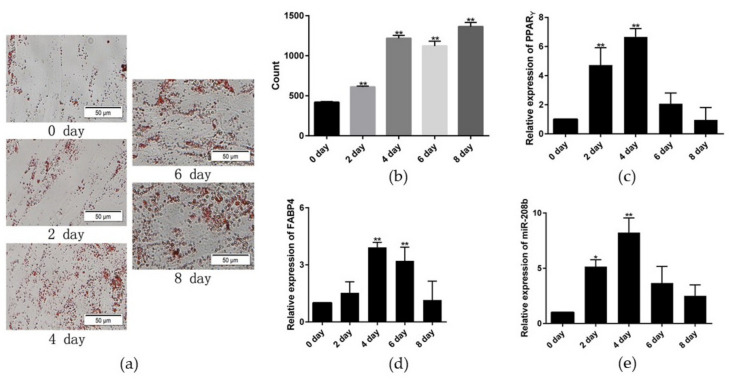
Establishment of rabbit preadipocyte differentiation model. (**a**) Oil Red O staining of lipid droplets at 0, 2, 4, 6, and 8 days of differentiation. (**b**) The number of lipid droplets (*n* = 3). (**c**,**d**) The relative expression levels of *PPARγ* and *FABP4* in rabbit preadipocyte after inducing differentiation at 0, 2, 4, 6, and 8 days. (**e**) The relative expression levels of miR-208b during preadipocyte differentiation progress. The data are presented as means ± SEM (*n* = 9). * *p* < 0.05; ** *p* < 0.01.

To establish a model of rabbit preadipocyte differentiation, the third generation of preadipocyte was cultured in cell culture plates and differentiated with the aforementioned method when the density reached 80%. Results of Oil Red O staining showed that the number of lipid droplets was significantly increased during the differentiation progress (Figure 1a,b). Simultaneously, preadipocyte differentiation key genes *PPARγ* and *FABP4* expression were significantly upregulated in the differentiation model and had the highest levels on day 4 (Figure 1c,d). Therefore, we conclude that the preadipocyte was successfully induced to differentiate. Besides, miR-208b expression was markedly different expression in the adipocyte differentiation model, revealing a possible regulatory role of miR-208b in preadipocyte differentiation (Figure 1e).

### 3.2. MiR-208b Promotes Rabbit Preadipocyte Differentiation

**Figure 2:**
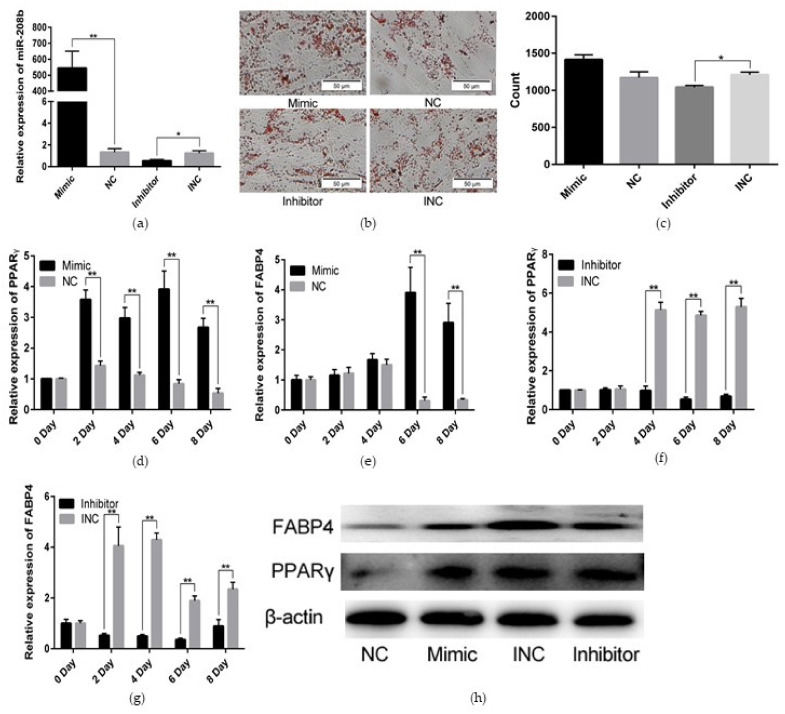
miR-208b promotes rabbit preadipocyte differentiation. (**a**) Transfection efficiency detection of miR-208b mimic and miR-208b inhibitor. (**b**) Oil Red O staining of lipid droplets after 8 days of transfection. (**c**) The number of lipid droplets (*n* = 3) after 8 days of transfection. (**d**,**e**) The relative expression levels of *PPARγ* and *FABP4* in rabbit preadipocyte induced differentiation at 0, 2, 4, 6, and 8 days after transfecting with miR-208b mimic and NC. (**f**,**g**) The relative expression levels of *PPARγ* and *FABP4* in rabbit preadipocyte induced differentiation at 0, 2, 4, 6, and 8 days after transfecting with miR-208b inhibitor and INC. (**h**) *PPARγ* and *FABP4* protein levels during preadipocyte differentiation after transfecting with NC, miR-208b mimic, INC, and miR-208b inhibitor. The data are presented as means ± SEM (*n* = 9). * *p* < 0.05; ** *p* < 0.01.

To assess the functional effect of miR-208b on rabbit preadipocyte differentiation, we transfected the miR-208b mimic, miR-208b inhibitor, NC, and INC into the preadipocyte, which was differentiated with the aforementioned method. As shown in Figure 2a. The relative expression levels of miR- 208b in the mimic (inhibitor) group were significantly higher (lower) than those in the NC (INC) group, indicating that transfection analogs successfully increased (decreased) the expression levels of miR-208b. Oil Red O staining showed that the number of lipid droplets was higher in the mimic group than the NC group, but the number of lipid droplets was lower in the inhibitor group than the INC group after 8 days of transfection (Figure 2b,c). Furthermore, we found an obvious and highly significant increase of genes in the mimic group, including *PPARγ* and *FABP4*, which are important transcription factors in the development and function of the adipose tissue and markers of preadipocyte differentiation (Figure 2d,e). In contrast, *PPARγ* and *FABP4* were expressed at lower levels in the inhibitor group (Figure 2f,g). We validated the qRT-PCR data by WB assays, using the total protein from the miR-208b mimic, miR-208b inhibitor, NC, and INC groups. We confirmed an increased expression of *PPARγ* and *FABP4* at the protein levels in the mimic group, but a decreased expression of *PPARγ* and *FABP4* in the inhibitor group (Figure 2h). Therefore, we concluded that miR-208b play a positive role in rabbit preadipocyte differentiation.

### 3.3. MiR-208b Promotes Rabbit Preadipocyte Proliferation

**Figure 3:**
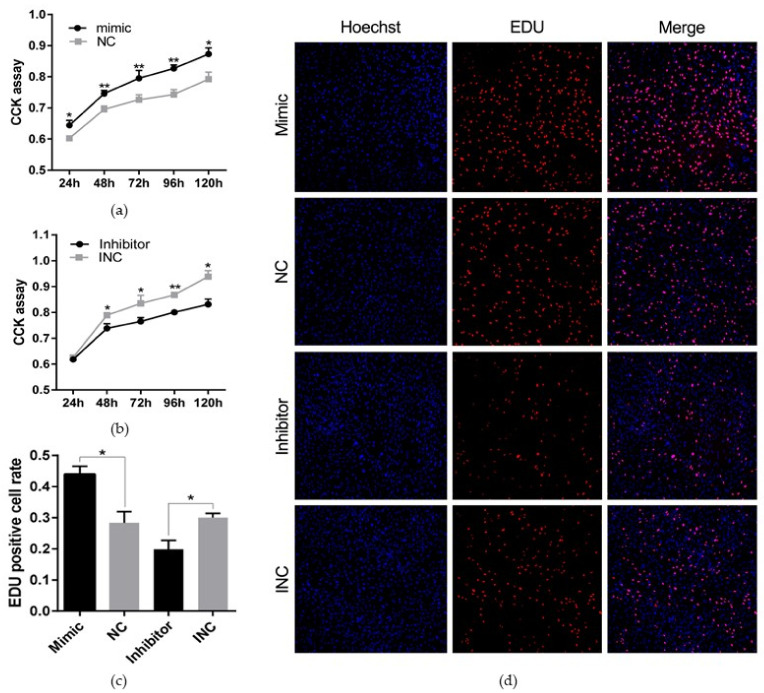
miR-208b promotes rabbit preadipocyte proliferation. (**a**,**b**) The absorbance of preadipocyte at 24, 48, 72, 96, and 120 h after transfection with the miR-208b mimic, NC, miR-208b inhibitor, and INC (*n* = 6). (**c**) The percent of EDU positive cells. Red fluorescence represents the EDU positive cells, and blue fluorescence represents the Hoechst stained cells. EDU positive cells rate = EDU positive cells/Hoechst stained cells × 100% (*n* = 3). (**d**) The picture of the EDU proliferation assay for preadipocyte transfected with the miR-208b mimic, NC, miR-208b inhibitor, and INC (red fluorescence represents EDU positive cells and nuclei are indicated by blue fluorescence). The data are presented as means ± SEM. * *p* < 0.05; ** *p* < 0.01.

We performed CCK and EDU proliferation assay by transfecting the miR-208b mimic, miR-208b inhibitor, NC, and INC into the preadipocyte cultured in vitro to assess the effect of miR-208b on rabbit preadipocyte proliferation. As shown in Figure 3a,b, the miR-208b mimic significantly increased the absorbance of preadipocyte after incubation for 24 h (*p* < 0.05), 48 h (*p* < 0.01), 72 h (*p* < 0.001), 96 h (*p* < 0.001), and 120 h (*p* < 0.05), but the miR-208b inhibitor significantly decreased the absorbance of the preadipocyte after incubation for 48 h (*p* < 0.05), 72 h (*p* < 0.05), 96 h (*p* < 0.001), and 120 h (*p* < 0.05). Besides, the EDU proliferation assay revealed that the number of positive cells was significantly higher in the mimic and INC groups than the NC and inhibitor groups, respectively (Figure 3c,d). These data suggest that miR-208b play a positive role on rabbit preadipocyte proliferation.

### 3.4. CSNK2A2 Is One of the Target Genes of miR-208b

**Figure 4:**
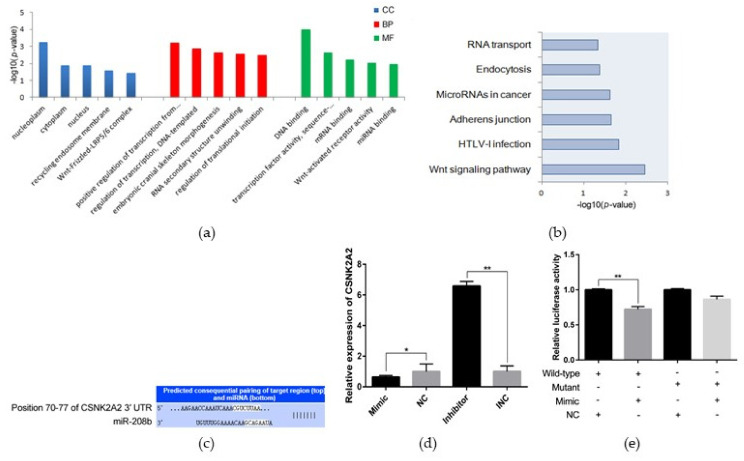
*CSNK2A2* is one of the target genes of miR-208b. (**a**) GO analysis of miR-208b target genes, only showing the top 5 significantly GO terms in BP, CC, and MF (*p* < 0.05). (**b**) The significantly enriched pathway of miR-208b target genes by using the KEGG database (*p* < 0.05). (**c**) The predicted binding site of gene *CSNK2A2* with miR-208b. (**d**) *CSNK2A2* mRNA levels after transfecting with the miR-208b mimic, NC, INC, and miR-208b inhibitor 24 h. (**e**) Luciferase assays were performed by cotransfection of wild-type or mutant plasmids with a miR-208b mimic in Hela cell, and the NC group was used as the control group (*n* = 3). The data are presented as means ± SEM (*n* = 9). * *p* < 0.05; ** *p* < 0.01.

To identify target genes that bind with miR-208b, we used online software TargetScan to perform target genes prediction, and results showed that 208 genes are the most likely candidates (Table S1). GO analysis found that a total of 62 enriched GO terms (33 biological processes (BP), 12 cellular components (CC), and 17 molecular functions (MF)), and 34 out of 62 GO terms (54.84%) were significantly enriched with *p* < 0.05 (Table S2). The enriched GO terms mainly included positive regulation of transcription from RNA polymerase II promoter (GO: 0045944), regulation of translational initiation (GO: 0006446), and translation initiation factor activity (GO: 0003743) (Figure 4a). Moreover, target genes were enriched in signaling pathways such as the Wnt signaling pathway, adherens junction, etc. (Table S3; Figure 4b). MiRWalk database revealed that *CSNK2A2* contains a potential binding site for miR-208b (Figure 4c). This observation was validated by a qRT-PCR assay and a luciferase reporter assay. *CSNK2A2* was significantly down-regulated in the miR-208b mimic group but upregulated in the inhibitor group in qRT-PCR analysis (Figure 4d). Moreover, luciferase reporter assay revealed that the luciferase activity was highly suppressed in the group containing the wild-type 3′ UTR of *CSNK2A2* mRNA but not significantly changed in the mutant group (Figure 4e). These observations imply that *CSNK2A2* is a direct miR-208b target.

## 4. Discussion

Currently, obesity has attained the degree of an epidemic, and the prevention and treatment of obesity have been unsuccessful [17]. Adipogenesis is a central event in the process of obesity. Importantly, cell proliferation and differentiation are key processes during adipogenesis and can be regulated by multiple factors [18]. The importance of miRNA was demonstrated in this pathological process [19,20]. Recent findings have found that miR-208b is an important factor and responsible for some diseases including chronic chagas disease cardiomyopathy, acute myocardial infarction, and metabolic syndrome [13,15,21]. However, the role of miR-208b in preadipocyte proliferation and differentiation has not been carried out. Thus, the detailed information was investigated in preadipocyte to better understand miR-208b-related function.

Hormones and nutrients are necessary for preadipocyte to transform into mature adipocyte capable of lipid synthesis, storage, and production of adipokines [4]. In our study, we applied exogenous drugs DEX, IBMX, and insulin to induce preadipocyte differentiation. *PPARγ* and *FABP4* are critical transcription factors in the development and function of the fat tissue and markers of preadipocyte differentiation [22,23]. The overexpression of PPARγ in preadipocytes leads to differentiation into mature adipocytes with adipogenic properties and adipokine expression [24]. Moreover, transcription factor *FABP4* is sufficient to induce preadipocyte differentiation and is correlated with markers of metabolic syndrome and related disease [25,26]. Here, in all tasted in vitro cell samples, qRT-PCR revealed that *PPARγ* and *FABP4* expression were detected and upregulated in the differentiation process, which was consistent with the previous finding [16]. Besides, we also observed different lipid drops by Oil Red O staining assay, compared with results from the qRT-PCR trial, indicating that preadipocyte was successfully induced to differentiate. Meanwhile, miR-208b level during the differentiation model reveals a possible regulatory role of miR-208b in preadipocyte differentiation.

In humans and animals, white fat tissue is a multifunctional organ supporting triglyceride storage for energy demands. Detailly, intracellular triglyceride is mainly derived from circulating non-esterified fatty acids and/or through hydrolysis of lipoproteins. As the precursor of mature adipocyte, preadipocyte is capable of proliferation and differentiation and these two processes are happened in quick succession [27]. Moreover, the number of adipocytes in white fat tissue mainly depends on the number of preadipocyte, endocrine, and dietary stimuli [28]. Thus, the proliferation and differentiation of preadipocyte are contributed to adipose tissue development and even body weight gain. In the present study, we found that upregulation of miR-208b increased the expression of *PPARγ* and *FABP4*, but downregulation of miR-208b reduced the expression of *PPARγ* and *FABP4* at both mRNA and protein levels. Moreover, the number of lipid droplets was found to be higher after transfection of miR-208b. These results suggest that miR-208b promotes the differentiation of preadipocyte and accumulation of intracellular triglyceride. Moreover, to intuitively determine the functional consequences of miR-208b in the proliferation of preadipocyte, we performed EDU and CCK analysis and found that miR-208b not only positively regulates the differentiation of preadipocyte but also enhance the proliferation of preadipocyte. Interestingly, a previous study revealed that miR-208b accelerates the proliferation and inhibits the differentiation of myogenic cells by targeting *TCF12* [29]. Thus, we conclude that miR-208b play different roles in different cells.

miRNAs are endogenous small RNA that play an important gene-regulatory role in animals by pairing to the mRNA of protein-coding genes to direct their post-transcriptional repression [10]. Here, a total of 208 target genes were identified using the online software TargetScan and were further analyzed with GO and KEGG to obtain an overview of these target genes and to further explore the function of miR-208b. GO and KEGG are convenient tools for understanding the biological function of genes and gene products [30]. The significantly enriched GO terms in the BP, CC, and MF revealed the possible function of miR-208b target genes in regulating adipogenesis. For example, regulation of translational initiation (GO: 0006446), transcription coactivator activity (GO: 0003713), translation initiation factor activity (GO: 0003743), and regulation of transcription, DNA-templated (GO: 0006355) were significantly enriched. Adipogenesis is a complex process in which pluripotent mesenchymal stem cells differentiate into mature adipocytes and is controlled by a series of regulators [31,32]. Regulating translation during protein synthesis is important for cell proliferation and differentiation [33]. In our KEGG pathway analysis, five pathways were significantly enriched, including the Wnt signaling pathway, adherens junction, etc. Wnt signaling is a molecular switch that controls adipogenesis. Wnt signaling maintains preadipocytes in an undifferentiated state through inhibition of the adipogenic transcription factors C/EBPα and PPARγ [34]. Overall, the Wnt signaling pathway is a negative regulator in adipogenesis when it is activated [35]. Moreover, a major membrane structure to connect cells is adherens junction (AJ), which comprises cadherin receptors, associated proteins termed catenins and cytoskeletons [36]. Dynamic changes in cell adhesion are needed to resolve and establish new cell contacts during developmental cell movements, tissue renewal, and wound repair [37]. Cytoskeletal remodeling and cell–cell interaction are necessary steps in the transformation of preadipocytes into mature adipocytes [38].

## 5. Conclusions

In conclusion, we applied exogenous drugs DEX, IBMX, and insulin to induce preadipocyte differentiation in vitro. We found that miR-208b was differently expressed during the progress of differentiation. Functionally, miR-208b positively promoted the expression of preadipocyte differentiation key genes *PPARγ* and *FABP4* at mRNA and protein levels. Both EDU and CCK analysis suggested that miR-208b was associated with preadipocyte proliferation. Overall, our data reveal that miR-208b play a positive role in preadipocyte differentiation and proliferation and may contribute to the prevention and treatment of obesity. However, there are some shortcomings in this study given various constraints. For example, among 208 putative genes, we only focused on *CSNK2A2* because it is a cellular activity regulator that is involved in multiple pathways such as the Wnt signaling pathway and adherens junction. The above two pathways are known pathways that regulate the function of preadipocyte. Moreover, given that cell activity is a complex process associated with genes, proteins, metabolites, etc., the candidate genes are also critical and should be taken seriously. Moreover, we did not use a different database to verify the analysis results, only one database cannot cover every aspect of the information. Thus, the further functional verification of miR-208b and miR-208b-target genes network will be important to consider in the future.

## Figures and Tables

**Table 1:** The sequence information of RNA oligo.

Name	Sequence Information (5′-3′)
miR-208b mimic	F:AUAAGACGAACAAAAGGUUUGU R:ACAAACCUUUUGUUCGUCUUAU
miR-208b inhibitor	ACAAACCUUUUGUUCGUCUUAU
miR-208b NC	F:UUGUACUACACAAAAGUACUG R:GUACUUUUGUGUAGUACAAUU
miR-208b INC	CAGUACUUUUGUGUAGUACAA

**Table 2:** The primer sequences were used for the qRT-PCR analysis.

Name	Forward Primer (5′-3′)	Reverse Primer (5′-3′)
*PPARγ*	GAGGACATCCAGGACAACC	GTCCGTCTCCGTCTTCTTT
*FABP4*	GGCCAGGAATTTGATGAAGTC	AGTTTATCGCCCTCCCGTT
*GAPDH*	CTTCGGCATTGTGGAGGG	GGAGGCAGGGATGATGTTCT
*CSNK2A2* miR-208b	GTGCTCTCCAGTGGTCTCAC ATAAGACGAACAAAAGGTTTGT	GGACAACAGGAACCGACCAT mRQ 3′ primer
U6	GGAACGATACAGAGAAGATTAGC	TGGAACGCTTCACGAATTTGCG

## Data Availability

All data generated or analyzed during this study are included.

## Supplementary Materials

The following are available online at https://www.mdpi.com/article/10.3390/genes12060890/s1, Table S1: The possible target genes of miR-208b, Table S2: The GO analysis result of miR-208b target genes, Table S3: KEGG pathways analysis of miR-208b target genes.
